# Online Extrinsic Calibration of Camera and LiDAR Based on Cascade Optimization

**DOI:** 10.3390/s26072282

**Published:** 2026-04-07

**Authors:** Chuanxun Hou, Zheng He, Tong Zhao, Zhenhang Guo, Xinchun Ji

**Affiliations:** 1 Rocket Force University of Engineering, Xi’an 710025, China; houcxun@sina.com (C.H.); 2021226056@chd.edu.cn (T.Z.); 2 University of Chinese Academy of Sciences, Beijing 100094, China; 3 Aerospace Information Research Institute, University of Chinese Academy of Sciences, Beijing 100094, China; guozh@aircas.ac.cn (Z.G.); jixc@aircas.ac.cn (X.J.)

**Keywords:** extrinsic calibration, feature classification, cascade optimization

## Abstract

**Highlights:**

**What are the main findings?**

- A two-stage camera–LiDAR extrinsic calibration algorithm is proposed to address the issues of initial value dependence and poor scene adaptability in existing targetless extrinsic calibration methods.
- To enhance the scene adaptability of the calibration algorithm, a cascaded optimization scheme of extrinsic parameter is designed in light of the varying constraint capabilities of edge features with different directions on individual extrinsic parameters.

**What are the implications of the main findings?**

- Cascaded optimization significantly reduces computational complexity compared to direct optimization, enabling fast and accurate extrinsic estimation.
- Compared to existing camera–LiDAR extrinsic calibration algorithms, the proposed algorithm achieves an accuracy advantage.

**Abstract:**

Accurate and stable extrinsic calibration is the foundation of high-quality fusion sensing and positioning of camera and Light Detection and Ranging (LiDAR). However, traditional targetless calibration methods suffer from limitations such as poor scene adaptability and unstable convergence, which significantly restrict calibration accuracy and robustness in complex environments. Aiming at solving those problems, we propose an online cascade-optimization-based extrinsic calibration method of combining motion trajectory alignment and edge feature alignment. In the initial calibration stage, a hand–eye calibration algorithm is designed by minimizing the residual discrepancies between camera odometry and LiDAR odometry sequences. It establishes a robust initialization for subsequent optimization. Then, in order to extract robust edge line features from sparse point clouds, we employ depth difference and planar edges of point clouds in the optimization process. Subsequently, principal component analysis (PCA) is applied to compute the principal direction of the extracted line features, enabling a decoupled optimization scheme that accounts for directional observability. This approach effectively mitigates the adverse effects of uneven environmental feature distributions. Experimental validation on typical urban datasets demonstrates the method’s generalizability and competitive accuracy: rotational parameter errors are constrained within 0.25°, and translational errors are maintained below 0.05 m. This affirms the method’s suitability for high-accuracy engineering applications.

## 1. Introduction

With the escalating complexity of application scenes, intelligent vehicles and other autonomous systems are increasingly compelled to employ multi-modal sensor configurations for achieving accurate and comprehensive environmental perception. They serve as a foundational prerequisite for implementing safe motion decision and control. The multi-modal sensor fusion technology effectively mitigates the inherent limitations of single-sensor systems, enabling complementary integration across heterogeneous data. Within this framework, the LiDAR–camera sensor ensemble stands as the preeminent configuration in intelligent transportation and robotic systems [1,2,3]. LiDAR provides precise 3D spatial target information with high ranging accuracy and robust immunity to illumination variations, yet it lacks color and texture detail. Conversely, cameras excel at capturing rich pixel-level features and semantic information, including target color and contour. But cameras suffer from inherent limitations in direct depth measurement and are susceptible to environmental variations such as illumination and weather conditions, leading to complex imaging dynamics and reduced stability. Therefore, LiDAR and cameras have strong complementarity, and the fusion of the two can simultaneously obtain spatial distance, geometric measurement, and semantic information so as to perceive the application scene with higher quality.

Critical to LiDAR–camera data fusion is the accurate and stable rigid body transformation between sensor coordinate systems, which can be represented by the rotation matrix and translation vector. It establishes the precise spatial correspondence between point cloud data and image pixels. According to whether a target is required, the LiDAR–camera’s extrinsic calibration can be divided into two categories: manual calibration and automatic calibration.

Manual calibration methods (also referred to as target-based calibration) utilize artificial markers with distinct geometric features and high-accuracy fabrication, such as checkerboards, triangular fixtures, and spherical targets. Zhang et al. [4] pioneered a calibration approach using planar checkerboard patterns viewed from unknown orientations, leveraging geometric constraints of coplanar points to solve the pose transformation. Subsequent studies addressing Zhang’s limitations focused on improving marker pose placement, constraint models, and geometric designs. ITAMI et al. [5] addressed checkerboard pose sensitivity by introducing point-plane constraints via LiDAR horizontal–vertical sampling. XIE et al. [6] enhanced Dhall’s method [7] of introducing point–line–plane constraints to improve calibration convergence and accuracy. Shi et al. [8] designed a hybrid ball-hollow plate marker using hollow plate edge features for initial estimation and projection iteration of spherical center for optimization, achieving 0.01 m translational and 0.07° rotational accuracy. Due to the high manufacturing accuracy of markers and the uniform distribution and easy extraction of artificial point, line, and plane features, manual calibration methods can achieve relatively high accuracy. Nevertheless, such methods necessitate the setup of dedicated calibration environments and exhibit significant reliance on manual intervention, rendering them unsuitable for online calibration scenes. This significantly diminishes the autonomy of unmanned vehicles and intelligent robots in complex operational environments.

Targetless methods do not require artificial targets and can extract point–line–surface scenes features or trajectory features in the environment to construct constraints for extrinsic calibration. Such methods can generally be classified into motion-based methods and feature-based methods. Motion-based methods leverage hand–eye calibration to match and align sensor trajectories, operating without requiring a common field of view between the camera and LiDAR. Nevertheless, their calibration accuracy remains limited by scale ambiguity in monocular vision systems and inherent errors in trajectory estimation, leading to suboptimal parameter estimation. Zhao Hang et al. [9] employed raw motion measurements combined with ground feature points and ground elevation constraints to estimate the 6-DoF extrinsic parameters of the LiDAR–camera system. The feature-based approach estimates extrinsic parameters by extracting and matching common-view features from the overlapping field of view between images and point clouds. Exploiting the ubiquity of edge information, Levinson [10] introduced edge mutual information between point clouds and images for calibration. Subsequent works by Yuan et al. [11] and Liu et al. [12] employed fixed-size and adaptive voxelization, respectively, to extract depth-discontinuous boundaries from building structured point clouds, achieving high-accuracy calibration. However, such methods require structured scenes to provide regular and sufficient structural features. Therefore, designing fast, accurate, and highly adaptable feature extraction algorithms is crucial for online feature-based calibration methods. In addition, these methods require relatively accurate initial extrinsic parameters to achieve rapid alignment between point cloud features and visual features, as well as fast convergence of extrinsic parameter optimization [13]. Additionally, deep learning-based approaches have increasingly been adopted for LiDAR–camera extrinsic calibration. Schneider et al. [14] pioneered the use of the RegNet network to achieve end-to-end estimation of 6oD pose transformation parameters. Xudong Lv et al. [15] proposed the self-supervised LCCNet model and achieved stable calibration results by matching the geometric structure and photometric consistency of point-cloud–image pairs. Deep learning-based methods, in general, are subject to the requirement of a large volume of training data while also exhibiting poor generalization ability and restrictive usage conditions.

To address the aforementioned challenges, this paper proposes an extrinsic calibration approach for LiDAR–camera systems based on the cascade optimization of motion trajectories and edge features. This approach enables the online estimation of extrinsic parameters in complex application scenes without the need for artificial markers. The main contributions of the proposed method are as follows:

(1) Based on the coarse-to-fine cascade calibration framework, a motion-trajectory-based calibration algorithm is proposed to enhance the constraint capability of trajectory consistency in scenes where camera scale is lost. And it provides a robust initial value of the extrinsic parameters for the calibration optimization stage.
(2) For weakly structured application scenes such as streets, a depth difference–planar edge joint feature extraction method is designed to process sparse point cloud data. It fully obtains multiple types of vertical and horizontal edge features such as tree trunks, streetlamp poles, walls, and shallow grooves on the road.
(3) The principal direction of the extracted features is calculated via PCA decomposition [16]. Considering the varying constraint capabilities of features with different principal directions on extrinsic parameters, cascaded optimization is adopted to achieve fast and accurate estimation.

## 2. Joint Calibration Based on Cascade Optimization

### 2.1. Basic Principle of Extrinsic Calibration

**Figure 1:**
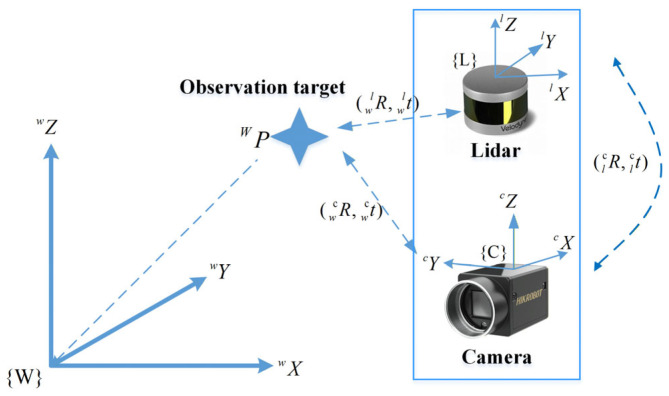
LiDAR and camera coordinate system conversion relationship.

The extrinsic calibration of the LiDAR and camera is essentially to solve the rigid body transformation matrix from the LiDAR coordinate system to the camera coordi-nate system, as shown in the Figure 1. The transformation matrix can be represented by a rotation matrix Rlc and a translation vector tlc.

Let PW be a point in three-dimensional space that is jointly observed by the camera and LiDAR. Pl=(Xl,Yl,Zl)T is the coordinate value of point PW in the LiDAR coordinate system and Pc=(Xc,Yc,Zc)T is the coordinate value of point PW in the camera coordinate system. Then there is

(1) Pc=Rlc⋅Pl+tlc

where Rlc∈SO(3), tlc∈ℝ3, and (⋅)lc represent the transformation from the LiDAR coordinate system to the camera coordinate system.

The camera data is usually represented in the form of an image, and the coordinate position of the spatial point Pc in the image is in pixels. The mapping from the three-dimensional point Pc in the camera coordinate system to the two-dimensional planar point pm in the pixel coordinate system can be realized using the camera’s intrinsic matrix K. In the three-dimensional to two-dimensional transformation process, the distance scale information is lost, that is,

(2) s⋅pm=K⋅Pc=K⋅(Rlc⋅Pl+tlc)

where pm=(u,v,1)T is the two-dimensional pixel point in the pixel coordinate system and s is the scale information.

### 2.2. Online Calibration in the Real Environment

**Figure 2:**
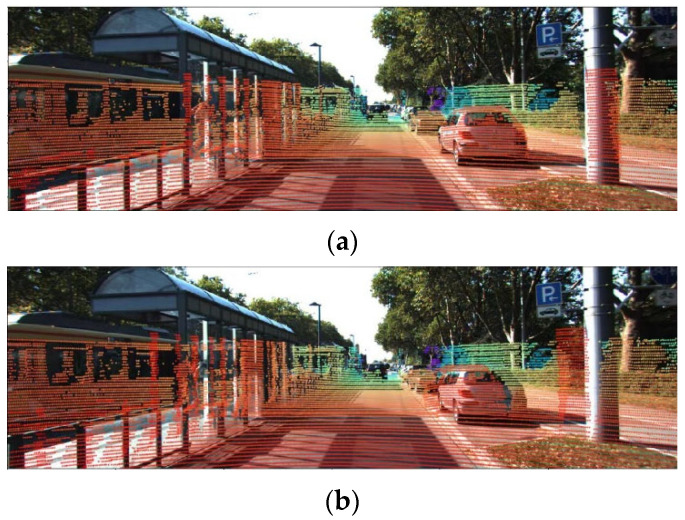
Project point cloud onto the image. (**a**) Accurate parameters by factory calibration. (**b**) Sensor pose changes cause parameter changes.

In the field of intelligent driving and autonomous robotics, the core connotation of online calibration is that calibration can be automatically performed during vehicle operation in natural environments such as open roads without human intervention, dedicated sites, or calibration targets. In the dynamic and complex real-world environments such as a typical urban street, it is impractical to pre-deploy artificial calibration features in the surroundings. This renders manual calibration or target-based calibration methods infeasible for online implementation, whereas the targetless extrinsic calibration scheme for LiDAR-camera systems has been validated to exhibit sufficient reliability [9,17]. Accurate extrinsic parameters can be obtained using co-observed specialized calibration devices, which are commonly employed in factory settings for unmanned vehicles and intelligent robots. In addition to this, during operational use, external disturbances such as road vibrations or impacts may alter the transformation matrix between LiDAR and the camera. In such cases, the pre-calibrated extrinsic parameter matrix no longer aligns with the current sensor transformation relationship, leading to misalignment between the camera and LiDAR coordinate systems. This misalignment further results in deviations when projecting point cloud data onto images, as illustrated in Figure 2.

**Figure 3:**
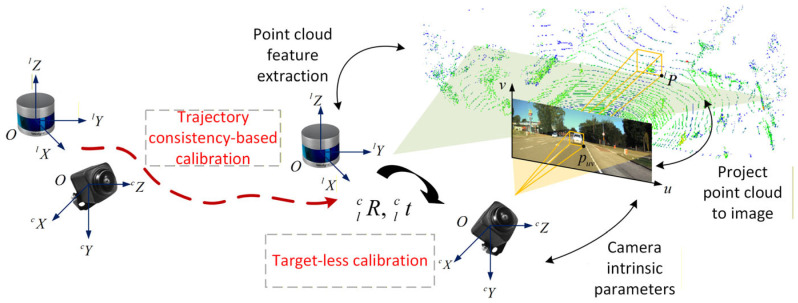
Schematic diagram of online feature-based extrinsic calibration of camera and LiDAR.

Under such circumstances, re-calibration of the LiDAR and camera is necessary to reinstate consistent environmental feature observations across sensors. However, target-based calibration methods depend on specialized calibration setups and entail time-consuming operational procedures. As a result, they are poorly suited for online calibration scenarios that require real-time adaptability. In contrast, targetless calibration schemes offer distinct application advantages for online implementation, with their operational workflow illustrated in Figure 3.

The feature-based targetless calibration method needs to identify the co-observed features of the image and the point cloud to solve the extrinsic parameters. Co-observed features include motion characteristics and environmental elements such as points, lines, and planes. The motion-based calibration method inversely solves the calibration parameters through a series of movement sequences of the sensor, but the calibration accuracy is usually not high due to the limitation of trajectory error. Motion-based calibration methods acquire motion sequences using the odometry of each sensor separately. They inversely solve the calibration parameters through the alignment of these motion sequences via the Iterative Closest Point (ICP) [18] or Similarity Transformation of Three Pairs (Sim(3)) [19] matching algorithm. However, due to inherent drawbacks of odometry such as trajectory drift, the calibration accuracy of such methods is generally unsatisfactory. The calibration algorithm based on environmental features constructs geometric constraints through the common view features to solve the transformation relationship between the two sensors. This algorithm exhibits strict dependencies on the initial extrinsic parameters, calibration scene characteristics, and spatial distribution of environmental features, thereby leading to inherent robustness limitations in diverse operational scenes.

In summary, the two aforementioned methods each exhibit complementary advantages in dynamic and complex real-world environments. On this basis, a cascade calibration algorithm can be designed. Specifically, the algorithm leverages a motion-based method to provide initial extrinsic parameter estimation in real time. Subsequently, it adopts the feature-based method to conduct precise estimation of the extrinsic parameters. The cascade framework ultimately achieves online targetless extrinsic calibration with high accuracy and robustness.

## 3. Methodology Design

### 3.1. Overall Process of the Methodology

The calibration of cascade optimization integrates motion trajectory alignment and edge feature matching.

**Figure 4:**
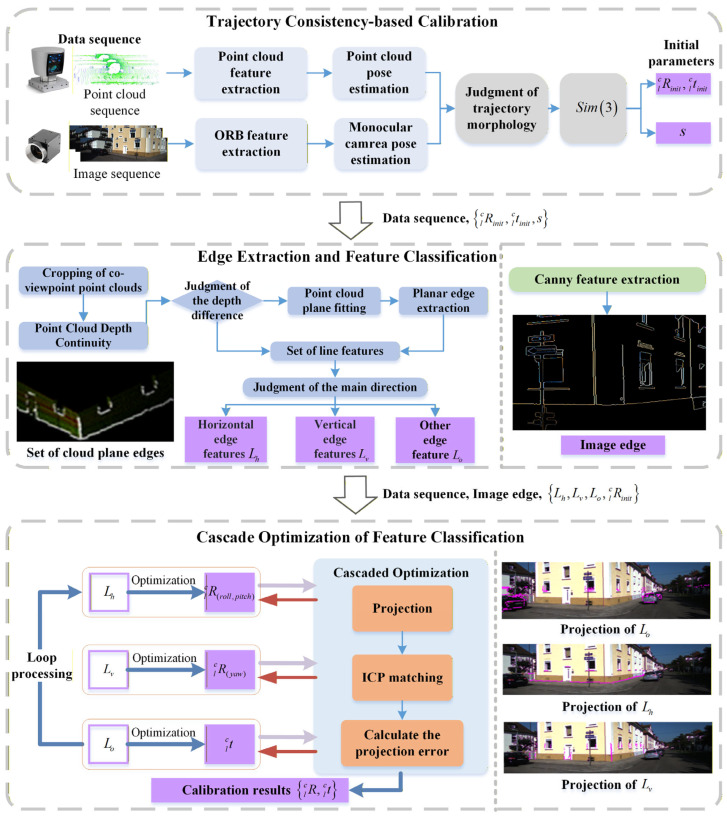
Processing flowchart of LiDAR and camera cascaded optimization online calibration algorithm. Blue boxes and lines represent the data processing flow, orange ones denote matching and optimization, and purple ones indicate the results of each module and extrinsic parameters to be optimized.

The proposed algorithm addresses two key limitations of existing algorithms, namely, suboptimal scene adaptability and convergence instability. Specifically, it employs a coarse-to-fine calibration strategy for varying adaptability scenes. Meanwhile, it implements cascade optimization on the decoupled rotation and translation parameters using different types of feature pairs to mitigate convergence instability. The detailed algorithmic workflow is depicted in Figure 4.

The calibration workflow primarily consists of three key modules: initial calibration based on motion trajectories, edge extraction and feature classification, and cascade optimization on the decoupled parameters relying on different types of features. In the initial calibration, the LiDAR’s motion trajectory is calculated by the lightweight LeGO-LOAM [20], and ORB-SLAM2 [21] is used to calculate the visual odometry to obtain the motion trajectory without scale. After assessing the trajectory morphology, the initial extrinsic parameters are determined by minimizing the trajectory matching residuals. In image edge extraction, an enhanced Canny operator is employed to generate a binary image that solely retains prominent edge information. For edge extraction from low-resolution point clouds, a joint extraction method combining depth differences and planar edges is adopted. It effectively enhances the recognition efficiency of building edges with subtle curvature, such as windowsills and corners. Finally, a cascaded optimization method for decoupling extrinsic parameters is proposed to further mitigate the impact of the uneven distribution of edge features on the convergence stability of the extrinsic parameters. It achieves fast and precise extrinsic calibration by utilizing the constraint capability of directional features in different orientations on extrinsic parameters.

The proposed algorithm is designed to meet the demand for real-time online application of camera–LiDAR calibration in vehicle-mounted intelligent perception scenarios. Its adaptability and automation run through the entire calibration pipeline, which is highly consistent with the engineering requirements of online calibration.

- Data processing: The algorithm does not require offline or pre-processing of perception data. Relying on the real-time frame streams captured by the camera and LiDAR, it can perform end-to-end incremental processing including trajectory extraction, edge feature detection and matching, and extrinsic parameter optimization, enabling calibration to proceed synchronously with data acquisition.
- Execution logic: The algorithm adopts a targetless automatic calibration framework that requires neither human intervention nor a dedicated calibration environment. It can be triggered automatically when the trajectory variation evaluation passes, without stopping the system for calibration, thus adapting to the dynamic operating characteristics of vehicle-mounted systems.
- Algorithm design: The cascaded optimization framework decomposes the original high-dimensional coupled extrinsic optimization problem into low-dimensional subproblems, which significantly reduces the computational complexity of core operations such as Jacobian inversion and Hessian matrix decomposition. Meanwhile, operations including point cloud field-of-view cropping and image HSV filtering achieve lightweight computation for the feature extraction module, allowing for the algorithm to converge rapidly on conventional hardware.

The above characteristics in terms of data stream processing, execution logic, and algorithm design together endow the proposed algorithm with the core attribute of real-time online calibration, which can effectively satisfy the dynamic online recalibration demand of vehicle-mounted sensors for intelligent driving.

### 3.2. Trajectory-Consistency-Based Calibration

The LiDAR and the camera are installed on the same movable platform. The initial extrinsic parameters (Rlcinit,tlcinit) can be calculated by aligning the trajectory information of the two at the same time stamp.

The lightweight LeGO-LOAM for ground vehicle applications is used to obtain the LiDAR trajectory. LeGO-LOAM calculates the frame-to-frame pose transformation by segmenting the ground point cloud and matching the plane features of the ground point cloud with the edge features of the non-ground point cloud in steps. If the angle between the vector formed by two points in the point cloud and the horizontal plane is very small (at approximately 10°), it can be approximately considered a ground point. Let the two points be pli=xli,yli,zli and plj=xlj,ylj,zlj; then the angle calculation is

(3) η=tan(zli−zlj)2(xli−xlj)2+(yli−ylj)2

where η is the angle between the horizontal plane and the vector composed of pli and plj.

After the ground point segmentation, the non-ground points are further clustered, and the simple and effective filtering process is realized by removing the smaller point cloud clusters. The plane features of the ground point cloud and the edge features of the non-ground point cloud are selected according to the curvature. Taking the point pli to be selected in the point cloud frame as the center, a certain number of points around pli are taken to form a set Nli to calculate the curvature cur:

(4) cur=1Nli⋅pli2∑j∈Nli∑j≠i(pli−plj)2

where $\left|\cdot \right|$ counts the number of points in the set Nli.

The points with large curvature values are classified as edge features, and the points with small curvature values are classified as plane features. The feature matching process is essentially a projection process. Specifically, the relative pose parameters (Rlk,tlk) of the k-th frame with respect to the previous frame are estimated by minimizing the point-to-line (edge) residuals and the point-to-plane residuals, as follows:

(5) J(Rlk,tlk)=minRlk,tlk∑i∈edge featureDie+∑i∈plane featureDipn

where $D_{i}^{e}$ and $D_{i}^{pn}$ represent the residual distances from the point-to-line and the point-to-plane, respectively. i denotes the index of the residual distance. Based on (Rlk,tlk), the k-th frame pose can be calculated by combining the historical trajectory points:

(6) plk=Rlk⋅plk+tlk

where Plk−1 is the point cloud odometry trajectory point of the (k−1)-th frame.

The ORB-SLAM2 [21] is used to obtain the camera trajectory. The camera odometry is calculated around the ORB features, combining the accuracy and speed advantages of the FAST key points and BRIEF descriptors. After the feature point matching, the Versatile Perspective-n-Point (VPnP) [22] problem is formulated to estimate the motion of the camera. Assuming that the i-th matched feature point pair is $x_{i}={\left[u_{i},v_{i},1\right]}^{T}$ and $x_{i}^{\prime }={\left[u_{i}^{\prime },v_{i}^{\prime },1\right]}^{T}$ and E is the essential matrix containing the rotation and translation information,

(7) E=tc^⋅Rcxi′TExi=0

where Rc and tc denote the rotation and translation transformation parameters of the current frame relative to the previous frame and the symbol (⋅)^ represents the skew-symmetric matrix.

The essential matrix E is solved according to the pixel positions of the matched point pairs, and the initial estimates of the camera motion Rci and tci are obtained by singular value decomposition (SVD). The initial estimates are optimized by constructing the minimization of the reprojection error problem of the feature points. ξck is the Lie algebra representation of the k-th frame camera pose transformation. Assuming that the spatial point coordinate set of k-th frame is {Pck,i,i=1,2,…,Nck} and its projected pixel coordinate set is {Pck,i,i=1,2,…,Nck}, Nck is the feature point number and $s_{k}$ is the scale information; thus, the relationship between pmk and Pck can be expressed as follows:

(8) skpmk=Kexp(ξck^)pck

where K denotes the intrinsic matrix of the camera. Initial estimates of the camera pose are inherently erroneous, and observation noise exists in feature point measurements. Due to the existence of above error, the least squares method is adopted to solve for the optimal pose that minimizes the reprojection error, as follows:

(9) ξck∗=argminξ12∑i=1Nckpmi−1siKexp(ξck^)Pci22

where ξck∗ is the optimal estimate of the k-th frame camera pose transformation and the k-th frame camera pose can be obtained by combining the historical trajectory points. It should be noted that the normalization process in the calculation makes the translation parameter tc lack scale information.

After obtaining the trajectory sequences of the LiDAR odometry and the visual odometry, the extrinsic parameter matrix is obtained by performing Sim(3) matching on the trajectory points of the camera coordinate system and LiDAR coordinate system at the same time stamp. Unlike ICP matching, Sim(3) matching incorporates a scale factor within the similarity transformation framework, explicitly accounting for monocular vision’s scale ambiguity. The optimization objective function is

(10) J(Rlcinit,tlcinit,s)=minRlcinit,tlcinit,s∑k∈Pl∩PcPck−s⋅RlcPlk−tlc2

where {Pl} and {Pc} represent the trajectory point sequences of the LiDAR and the camera, respectively.

**Figure 5:**
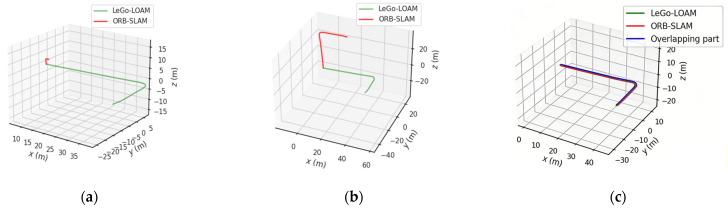
Trajectory-consistent initial extrinsic parameter calibration processing result diagram. (**a**) Initial trajectory sequence. (**b**) Camera sequence scale recovery. (**c**) Camera and LiDAR trajectory pose consistency.

The least squares method is used to solve the optimal value of the extrinsic parameters based on the trajectory consistency. The visual and LiDAR odometry have cumulative errors, and the motion trajectory with a single form (such as a straight line) will lead to the poor convergence accuracy of the rotation extrinsic parameters Rlc and unobservability of the translation parameters tlc. Therefore, it is imperative to evaluate the attitude variation in the trajectory to be calibrated, and the average attitude angle change in the window is used to evaluate the trajectory variation:

(11) $$trj=\max\left(0,\sum_{k\in (i,j)}\frac{\left|\Delta \theta_{k}\right|}{t_{i}-t_{j}}-\varepsilon_{trj}\right)$$

where $\Delta \theta_{i}$ is the attitude angle increment of the i-th frame. For the ground mobile platform, the pitch angle change can be selected, and $\varepsilon_{trj}$ is the cumulative attitude angle change threshold. The carrier executes a specific steering maneuver, with the $\varepsilon_{trj}$ set to 90 degrees. By comparing the average attitude angle change in the window with the threshold, it is judged whether the current trajectory contains a significant turning form so as to determine whether it can be used for extrinsic parameter calculation. Figure 5 illustrates the extrinsic parameter estimation process grounded in trajectory consistency, where the red and green lines denote the camera and LiDAR trajectory sequences, respectively.

As depicted, the original trajectories exhibit pronounced scale and attitude disparities due to the scale uncertainty of monocular vision and sensor-specific motion characteristics. Through Sim(3) similarity transformation, the two trajectory sequences are effectively overlapped without obvious trajectory distortion and deviation. By incorporating trajectory variation evaluation, the proposed method significantly strengthens the constraint on rotational parameters. Motion-based calibration is less susceptible to scene feature adaptability [9] and can provide reliable and robust initial estimates of extrinsic parameters for the subsequent fine-grained calibration stage in real time.

### 3.3. Edge Extraction in the Transform Domain

Edge extraction for images and point clouds constitutes a fundamental data processing step for edge matching in the calibration. The core objectives are to extract robust edge features from the environment and establish the spatial relative pose constraint between point cloud features and image features. Ultimately, the optimal estimation of the camera–LiDAR extrinsic parameters is obtained via spatial consistency-based accurate registration.

**Figure 6:**
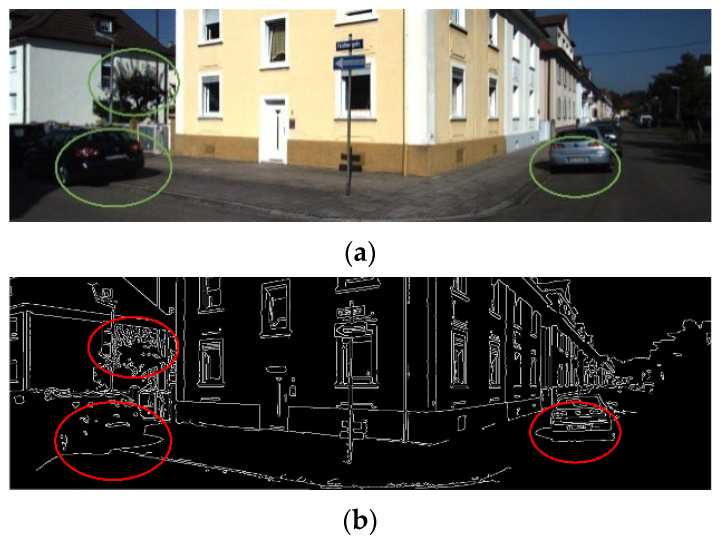
Interference of unstructured edges such as stray textures and shadows in the image. (**a**) Original image information of the calibration scene. (**b**) Edge feature extraction result of the Canny operator.

The well-established Canny algorithm [23] is commonly employed for image edge extraction. Due to the inherent characteristics of sensor data perception, the edges retrieved from images typically exhibit greater richness compared to the structural edges present in point clouds [24]. Moreover, these image edges are highly sensitive to elements such as stray textures and shadow edges in the scene. Due to factors such as illumination and shadows, images contain major irregular edges and stray textures, as shown in Figure 6. These edges interfere with the correlation of spatial structural information between images and point clouds, thereby degrading the performance of edge consistency alignment [24].

The HSV (Hue, Saturation, Value) threshold filtering method is used to reduce the influence of the above stray textures, shadows, and other irregular edges. The HSV color space decouples RGB information into three independent channels of hue (H), saturation (S), and value (V), which ensures the stability of color features in scenes with fluctuating illumination [25]. In addition, filtering on the S channel can eliminate interference regions with low saturation, such as the sky and white walls. The shadow and stray texture regions M are defined as follows:

(12) $$\begin{array}{c} M=\frac{\sigma_{s}S-\sigma_{v}V}{\sigma_{h}H+\sigma_{s}S+\sigma_{v}V}\\ if\left\{\begin{array}{l} M>\varepsilon_{S}\;\mathrm{area}\;\mathrm{in}\;\mathrm{shadow}\\ M\leq \varepsilon_{S}\;\mathrm{area}\;\mathrm{out}\;\mathrm{shadow} \end{array}\right. \end{array}$$

where $\sigma_{h},\sigma_{s},\sigma_{v}$ are empirical coefficients according to reference [25].

Threshold-based segmentation is performed on the HSV filtering result M to generate a mask. Subsequently, the Canny edges located within the mask region and four pixels of the outer contour are removed. This enables the more reliable extraction of image edge features and reduces the probability of mismatches in nearest neighbor association between 3D structural features and 2D image edges.

**Figure 7:**
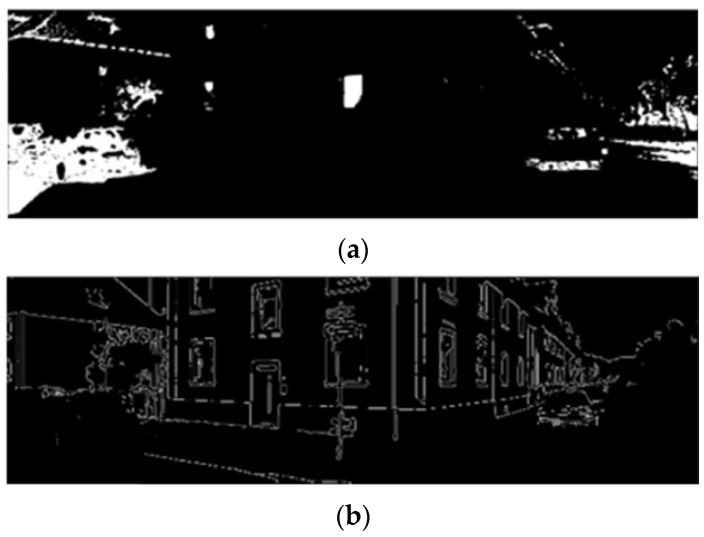
Image edge extraction result after HSV threshold filtering. (**a**) Threshold segmentation result. (**b**) Results of outer contour removal based on the segmentation results.

The edge extraction results are illustrated in Figure 7. Through image masking processing in the HSV color space, the interference of unstructured factors such as leaf and shadow boundaries on edge extraction is significantly reduced.

### 3.4. Point Cloud Edge Extraction and Classification

To improve the efficiency of point cloud edge feature extraction, the field of view (FOV) of the LiDAR is roughly aligned with that of the camera with the extrinsic parameter (Rlcinit,tlcinit). The alignment enables the cropping of point cloud data within the camera’s FOV, thereby effectively reducing the computational cost of point cloud edge feature extraction.

The point cloud is projected onto the two-dimensional image using (2). The homogeneous coordinates {(umi,vmi)|i=1,2,…} of the projected points on the image plane form a two-dimensional point set, denoted as {Qm}, and the point cloud outside the image field of view after projection is removed. The point set {Q⌣m} covered by the image is retained:

(13) {Q⌣m}∈{Qm | 0≤umi≤W,0≤vmi≤H}

where (W,H) is the pixel width and height of the image and i is the index of projected points. According to the point set {Q⌣m} within the image field of view, the corresponding three-dimensional point {Q⌣l} cloud can be indexed. In that case, the subsequent point cloud edge extraction is only for {Q⌣l}, reducing the computational cost and the interference of other point cloud edges outside the field of view.

The point cloud edges mainly include the object surface contour and the edge where the surfaces intersect. For the cropped point cloud, the vertical edges in the scene point cloud can be calculated using the depth discontinuity. The calculation of the point cloud depth difference is as follows:

(14) ∀ pli∈{Q⌣l},dli=0elsem¯lim¯li≥ρ

And normalized depth difference m¯l is

(15) m¯li=mli−min(mli)max(mli)−min(mli)mli=max(rli−1−rli,rli+1−rli,0)0.5

where rli represents the distance from the point pli to the LiDAR. The normalized depth difference m¯li is used as the edge attribute value dli of the point pli. ρ is the normalized depth difference threshold, which is usually set to one-fourth or one-third of m¯l. The points with non-zero edge attribute values dli are extracted as the edge point cloud set {Q⌣led}.

The depth discontinuity of the point cloud has an obvious effect on the edge feature extraction of slender objects in the scene, such as the regions with significant depth differences around lamp poles and tree trunks. In practical applications, due to the point cloud sparsity, it is difficult for the depth discontinuity method to robustly extract the edges of dense point clouds around corners and window frames. Considering above issue, the proposed method supplements the deficiency of edge extraction by extracting the plane edges of the point cloud. First, three non-collinear points (xl1,yl1,zl1),(xl2,yl2,zl2),(xl3,yl3,zl3) are randomly selected in the original point cloud to calculate the normalized equation of the corresponding plane:

(16) $$\left\{\begin{array}{l} A_{i}x_{l}+B_{i}y_{l}+C_{i}z_{l}+D_{i}=0\;(i=1,2,3)\\ A_{i}^{2}+B_{i}^{2}+C_{i}^{2}=1,\;D_{i}=1 \end{array}\right.$$

$\left(A_{i},B_{i},C_{i},D_{i}\right)$ denote the normalized equation parameters of the plane. The distance from the selected point cloud (xl,yl,zl) to the plane is calculated as follows:

(17) d=|Aixl+Biyl+Cizl+Di|Ai2+Bi2+Ci2   =|Aixl+Biyl+Cizl+Di|

Whether or not a point belongs to the plane is determined by its distance to the plane. The points within the allowable error range (the settings are as in [26]) are regarded as the inliers of the plane, and the number of inliers is counted. After multiple iterations, the plane parameters $\left({\hat{A}}_{i},{\hat{B}}_{i},{\hat{C}}_{i},{\hat{D}}_{i}\right)$ corresponding to the maximum inlier number are selected as the plane model. Finally, the inliers in the plane are removed from $\left\{{\overset{⌣}{Q}}_{l}\right\}$, and the remaining points are continuously fitted to extract other planes.

After obtaining all the plane point clouds, the Angle-Criterion [27] (AC) method is used for boundary extraction for each cloud. The AC method leverages the neighborhood property of edges, which implies that there necessarily exist other points belonging to the same edge in the neighborhood of an edge point and that the edge points within the same neighborhood are distributed exclusively on one side of the current edge point. To mitigate the bias of neighborhood centers toward high-density directions (caused by uneven point cloud distribution), a symmetry-based strategy is adopted to construct the kε-neighborhood.

First, the k-nearest points are searched around the center point, forming the main circular neighborhood point set $\left\{P_{p}^{k}\right\}$. Then, a neighborhood search using a circle with the radius ε is performed, and the resulting kε-neighborhood point set is denoted as $\left\{P_{p}^{k\varepsilon }\right\}$. Then, based on the relationship between adjacent neighborhood point sets, the neighborhood of the points in the region with a sharp change in point cloud density is also added to the neighborhood to form a symmetric kε-neighborhood:

(18) $$P_{p}^{sk\varepsilon }=\{q|p\in P_{q}^{k\varepsilon }\vee q\in P_{p}^{k\varepsilon }\}$$

**Figure 8:**
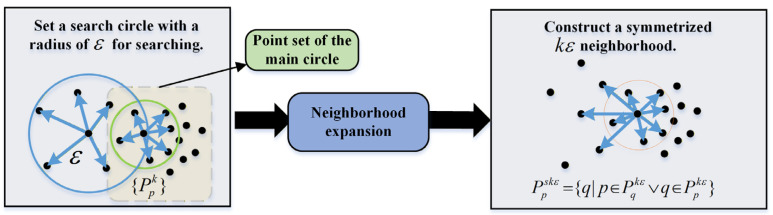
Neighborhood construction flowchart for AC edge extraction.

This means that when the point p (in the neighborhood $\left\{P_{p}^{k\varepsilon }\right\}$) belongs to the kε-neighborhood point of the point q (in the neighborhood $\left\{P_{q}^{k\varepsilon }\right\}$), q is regarded as the neighborhood point of p. The construction process is shown in Figure 8.

After constructing the KDTree neighborhood point set $\left\{P^{sk\varepsilon }\right\}$, the edge points are determined by the included angle criterion. For the neighborhood point set $\left\{P_{in}^{sk\varepsilon }\right\}$ corresponding to non-edge regions, the included angle distribution within this set is more uniform. In contrast, larger included angles are present in the neighborhood point set $\left\{P_{bo}^{sk\varepsilon }\right\}$ that belongs to edge regions. Taking the nearest neighbors of the point p in the point set $\left\{P_{bo}^{sk\varepsilon }\right\}$, connecting them pairwise with p forms a series of included angles $\Gamma =\{\gamma_{1},\gamma_{2},\ldots ,\gamma_{n}\},n=\left|P_{bo}^{sk\varepsilon }\right|-1$. The maximum value $\gamma_{\max}$ is found, and the probability that the point p is an edge point is calculated:

(19) $$\psi (p)=\min\left\{\frac{\gamma_{\max}-2\pi /\left|P_{p}^{sk\varepsilon }\right|}{\pi -2\pi /\left|P_{p}^{sk\varepsilon }\right|},1\right\}$$

where $p\in \{P_{bo}^{sk\varepsilon }\}$, and $\left|P_{bo}^{sk\varepsilon }\right|$ represents the point number of neighborhood point sets.

**Figure 9:**
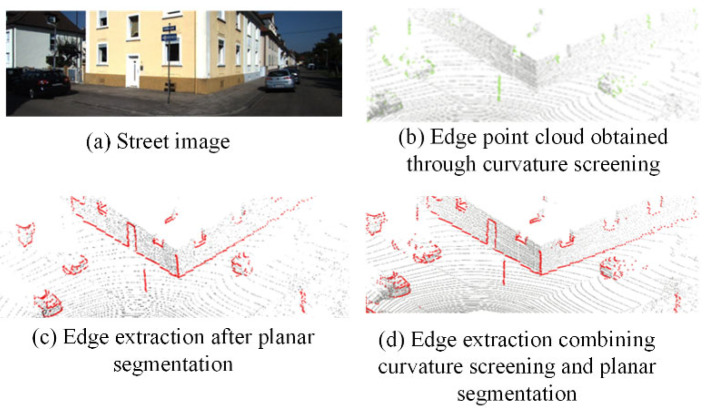
Edge extraction in the real scene.

The edge points obtained according to the probability can be divided into horizontal edge feature $L_{h}$, vertical edge feature $L_{v}$, and other edge feature $L_{o}$, as shown in Figure 9. According to the different constraints of the features on the pose elements, in the feature-matching optimization process, constraints are established for different extrinsic parameters using features with distinct directional distributions.

In the feature classification process, the edge lines are segmented via edge point clustering to construct KDTree for searching and processing on clustering region. For each cluster, the principal axis vector is obtained by principal component analysis (PCA), and the principal axis direction is the direction of the edge line. First, the point cloud is decentralized:

(20) $$\begin{array}{l} \tilde{P}=\left\{p_{0}-\bar{p},p_{1}-\bar{p},p_{2}-\bar{p},\ldots ,p_{n}-\bar{p}\right\}\\ \bar{p}=\frac{1}{n}\sum_{i=1}^{N}p_{i} \end{array}$$

The covariance matrix of the point cloud is calculated and the SVD is performed to determine the principal direction of the point cloud. The direction of the eigenvector corresponding to the maximum eigenvalue is selected as the principal direction of the point cloud:

(21) $$\begin{array}{l} H(x,y,z)=\left(\begin{array}{ccc} Cov(x,x) & Cov(x,y) & Cov(x,z)\\ Cov(y,x) & Cov(y,y) & Cov(y,z)\\ Cov(z,x) & Cov(z,y) & Cov(z,z) \end{array}\right)\\ \;\;\;\;\;\;\;\;\;\;\;\;\;\;\;\;\;\;\;\;=U_{r}\left(\begin{array}{ccc} \lambda_{1} & & \\ & \lambda_{2} & \\ & & \lambda_{3} \end{array}\right){U_{r}}^{T},\;\lambda_{1}\geq \lambda_{2}\geq \lambda_{3}\geq 0 \end{array}$$

Therefore, the principal direction vector s of the point cloud is equal to the unit eigenvector ${\left(U_{r}\right)}_{1:}/\left|{\left(U_{r}\right)}_{1:}\right|$ corresponding to $\lambda_{1}$. Thus, the angle $\theta =\arcsin\left(n\cdot s/\left|n\right|\cdot \left|s\right|\right)$ between the principal direction vector and the normal vector of the X–Y plane (the Z axis) can be calculated, where the normal vector denotes n=(0,0,1). When the angle between the principal axis and the normal vector is close to a right angle, the edge line is parallel to the X–Y plane and is recorded as a horizontal edge feature $L_{h}$. When the angle between the principal axis and the normal vector is close to 0, the edge line is parallel to the normal vector and is recorded as a vertical edge feature $L_{v}$. Then the remaining edge lines are recorded as other edge features $L_{o}$. The specific process is shown in Algorithm A1 in the Appendix A.

### 3.5. Cascaded Extrinsic Parameter Optimization

In the refinement calibration, a cascaded reprojection optimization method is proposed by leveraging the edge feature classification results. This method exploits the differential constraint capabilities of features with different principal directions on each dimension of rotation–translation parameters [11].

Firstly, coarse-matching performed on the other edge feature $L_{o}$ is used to roughly align the observation fields of view of the camera and the LiDAR, obtaining the optimized translation parameters. Other edge features incorporate non-directional and more uniformly distributed scattered point features into the construction of constraints. Positional offsets induced by position changes (i.e., translations) will significantly alter the relative pose of all objects in the scene within the observation field of view [28]. The positional offsets of other edge features can be clearly captured, thus yielding higher observability of matching residuals.

(22) J(Rlc,tlc)=mintlc∑i∈LoPci−Rlc(Pli)−tlc2

In the optimization of rotation parameters, horizontal edge features and vertical edge features are used to optimize the vertical-direction extrinsic parameters (yaw angle and roll angle) and horizontal-direction extrinsic parameters (pitch angle), respectively.

(23) J(Rlc,tlc)=minRlc(yaw,roll)∑iPci−Rlc(yaw,roll)(Pli)−tlc2,  i∈LvminRlc(pitch)∑iPci−Rlc(pitch)(Pli)−tlc2,  i∈Lh

Taking the horizontal edge features $L_{h}$ (where the length l in the horizontal direction is much greater than the width w in the vertical direction) as an example, when an equivalent observation angle deviation Δθ occurs in both the vertical and horizontal directions (i.e., vertical and horizontal residuals Δr arise), the relative change in the residual in the vertical direction (Δr/w) is much larger than that in the horizontal direction (Δr/l). This indicates that the parameters in the vertical direction (roll and yaw angle) are more sensitive to, and constrained by, horizontal features.

**Figure 10:**
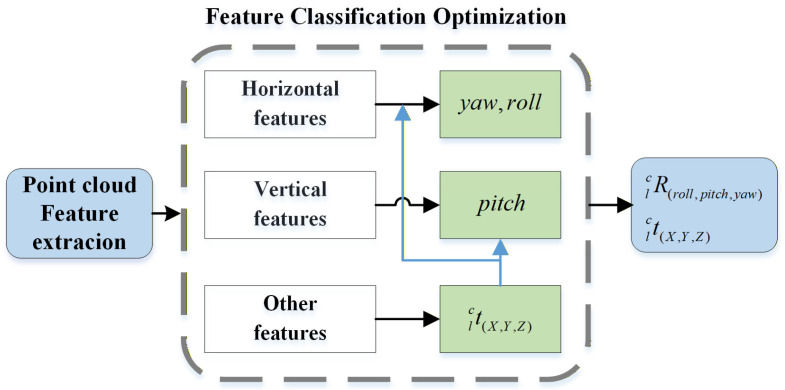
Cascaded optimization uses different principal orientation features to constrain extrinsic parameters in different directions.

The fine-calibration loop sequentially executes the cascaded optimization described in Equations (22) and (23), and finally obtains a stably converged estimation result of the extrinsic parameters. The feature cascaded optimization process is illustrated in Figure 10.

In engineering practice, the solution process of the optimization function composed of reprojection errors such as Equations (22) and (23) is divided into three stages: KD-Tree nearest neighbor search for feature pairs, iterative transformation solving, and transformation application. For an optimization problem with a total number of features $N_{L}$ (here, it is assumed that the number of image features and point cloud features are both $N_{L}$ for ease of calculation), the complexity of constructing a KD-Tree for the target point cloud in 3D space is $O(N_{L}\log N_{L})$ [29]. In a single iteration of transformation solving, the complexity of performing a nearest neighbor search for $N_{L}$ points via the KD-Tree is $O(N_{L}\log N_{L})$. Combined with the linear complexity $O(N_{L})$ of centroid calculation, SVD-based rigid transformation solving, and point cloud transformation, the total complexity of a single iteration is $O(N_{L}\log N_{L})+O(N_{L})\approx O(N_{L}\log N_{L})$. Let the number of iterations required for convergence be a constant $K_{d}$; then the total complexity of the entire process is $O(K_{d}N_{L}\log N_{L})$.

After feature classification, assuming the numbers of features in categories $L_{o}$, $L_{v}$, and $L_{h}$ are $N_{L_{o}}$, $N_{L_{v},}$, and $N_{L_{h}}$ respectively, satisfying $N_{L_{o}}+N_{L_{v},}+N_{L_{h}}<N_{L}$, the computational complexity of the KD-Tree nearest neighbor search and a single iteration is equal to $O(N_{L_{o}}\log N_{L_{o}})+O(N_{L_{v}}\log N_{L_{v}})+O(N_{L_{h}}\log N_{L_{h}})$, which satisfies

(24) $$\begin{array}{l} N_{L_{o}}\log N_{L_{o}}+N_{L_{v}}\log N_{L_{v}}+N_{L_{h}}\log N_{L_{h}}<N_{L}\log N_{L}\\ st.\left\{\begin{array}{l} N_{L_{o}}+N_{L_{v},}+N_{L_{h}}<N_{L}\\ N_{L_{o}}\in {\mathbb{N}}^{*},N_{L_{v}}\in {\mathbb{N}}^{*},N_{L_{h}}\in {\mathbb{N}}^{*} \end{array}\right. \end{array}$$

Since the objective function of low-dimensional subproblems is smoother, the coupling interference between parameters is eliminated, which effectively avoids iterative oscillation and reduces the number of iterations $K_{c}$ required for the convergence of cascaded optimization being much smaller than $K_{d}$ (i.e., $K_{c}\ll K_{d}$). Therefore, the computational complexity of cascaded parameter optimization $O[K_{c}(N_{L_{o}}\log N_{L_{o}}+N_{L_{v}}\log N_{L_{v}}+N_{L_{h}}\log N_{L_{h}})]$ is much smaller than that of direct optimization $O(K_{d}N_{L}\log N_{L})$. Furthermore, the feature classification optimization strategy has inherent parallel processing characteristics, exhibiting promising potential for parallel acceleration when deployed on vehicle-mounted embedded GPUs/AI chips. The three factors synergistically achieve a significant improvement in computational efficiency.

## 4. Experimental Verification

The online calibration algorithm proposed in this paper is verified with the data from the KITTI-2011-10-03-0027 dataset, S3E dataset, and TUMTraf A9 Highway Dataset. All experiments were conducted on the robot operating system (ROS) in Ubuntu 20.04, and computing platform was equipped with a 3rd Gen Intel(R) Core(TM) i9-13900H CPU and 32 GB RAM. The KITTI-2011-10-03-0027 dataset was employed for internal validation to verify the accuracy and stability of the proposed algorithm, as well as the effectiveness of the cascaded optimization. The S3E dataset [30] and TUMTraf A9 Highway Dataset [31] were used for horizontal comparison to evaluate the generalization ability and calibration accuracy against existing network-based and semantic segmentation-based methods.

**Figure 11:**
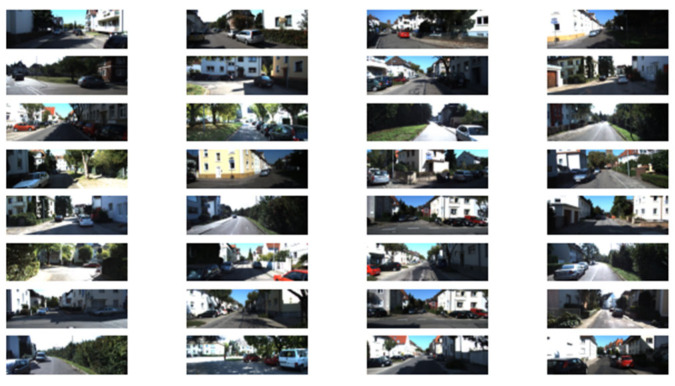
Various scenes of the KITTI dataset.

The total length of the KITTI-2011-10-03-0027 trajectory was 3744.0 m, and the continuous measurement time was 470.58 s. As shown in Figure 11, The overall environment had the following characteristics:

- This segment of data belonged to the “residential area” part. The vehicle speed was relatively low, and there are many sudden stops.
- There were many houses and trees of various shapes on both sides of the road, which was quite friendly for the selection and matching of image and point cloud feature points.
- During the vehicle’s driving process, due to the narrow road and the large number of pedestrians and vehicles, the LiDAR occluded quite frequently.

Due to the occlusion, there are obvious changes in light and darkness, and overexposure also exists in some sections.

Both the S3E and TUMTraf A9 Highway Dataset are widely used in the fields of vision/LiDAR scene perception, object recognition, and localization and navigation. They collect visual images and LiDAR point clouds via mobile UAVs/vehicles in typical urban driving scenarios such as traffic roads and urban blocks. In addition, the scenes contain numerous dynamic interfering objects, such as pedestrians and motor vehicles, which pose challenges to the robustness and generalization ability of the algorithm. Meanwhile, due to environmental factors, including tree shade and building occlusion, the illumination conditions in the scenes of S3E dataset undergo drastic dynamic variations. Since the TUMTraf A9 Highway Dataset introduces a novel observation paradigm where vehicles on the road are observed from a tilted downward perspective via traffic sign poles, the proposed algorithm initializes the rotation matrix as the identity matrix and the translation vector as the zero vector, and performs fine calibration directly.

In the accuracy evaluation of the experiments, mean squared error (MSE) was adopted to calculate the rotation error $error_{R}$ and translation error $error_{T}$. The formula is given as follows:

(25) $$\begin{array}{l} error_{R}=\sqrt{{\left(yaw-yaw_{gt}\right)}^{2}+{\left(pitch-pitch_{gt}\right)}^{2}+{\left(roll-roll_{gt}\right)}^{2}}\\ error_{T}=\sqrt{{\left(x-x_{gt}\right)}^{2}+{\left(y-y_{gt}\right)}^{2}+{\left(z-z_{gt}\right)}^{2}} \end{array}$$

where (yaw,pitch,roll,x,y,z) denote the calibration results and $\left(yaw_{gt},pitch_{gt},roll_{gt},x_{gt},y_{gt},z_{gt}\right)$ denote the ground-truth values.

### 4.1. Trajectory-Consistency-Based Calibration Results

**Figure 12:**
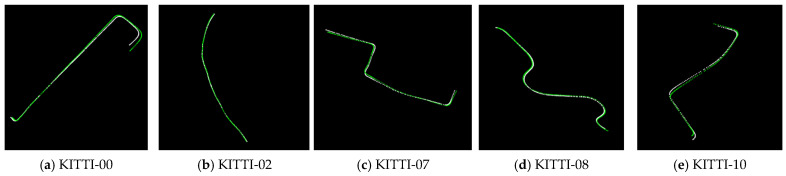
Trajectory-matching results of visual odometry and LiDAR odometry. By comparing the five groups of trajectory-matching results, it can be seen that the scale of the visual odometry is accurately restored and two groups of odometry trajectories can be accurately overlapped without significant trajectory divergence.

The selected data are subjected to hierarchical coarse-matching and fine-matching. First, the LiDAR odometry trajectory and visual odometry trajectory of five selected data were obtained using the algorithms LeGO-LOAM and ORB-SLAM2. The trajectory-consistency-based calibration was performed on the odometry trajectory data of the selected road section. The trajectory-matching results are shown in Figure 12.

Meanwhile, the initial calibration quantitative analysis was conducted, as shown in Table 1. The pose initialization by the initial trajectory alignment method has an error between 1° and 1.5° compared to the ground-truth, which can provide a reliable initial value for fine calibration.

### 4.2. Accuracy of Distributed Reprojection

After obtaining the initial extrinsic parameters, the edge image and edge point cloud are registered by the cascaded optimization. To verify the effectiveness of the proposed optimization method, the direct optimization method (the rotation and translation parameters are optimized simultaneously) is used for comparison on the same data. The accuracy comparison results are shown in Table 2 (averaged for three scenes in each sequence), which describes the deviation degree of calibration results from the true values.

The results in Table 2 show that, compared to the direct reprojection method, the calibration accuracy of the cascaded optimization method has a higher accuracy in each dimension of the rotation error. The overall accuracy can reach that each rotation parameter error is better than 0.25°, and the translation parameter error is better than 0.04 m.

### 4.3. The Repeatability and Consistency

To assess the consistency of the algorithm, the repeatability of the calibration re-sults within the same scene, as well as the consistency of the calibration results across different scenes within the same sequence and among different sequences, will be se-quentially evaluated, as shown in Table 3. 

Taking the frame 000102 in scene 01 as an illustrative case for repeatability verification, the results of multiple experiments consistently exhibit fluctuations around the mean value, with the errors being less than (0.03°, 0.01 m). No outlier singular values are observed, indicating a high level of overall consistency. Subsequently, the deviation values of the calibration result across different scenes within the same sequence are compared. Taking scene 00 as an example, the corresponding results are presented in Table 4.

Repeated calibration experiments were conducted to demonstrate the stability of the algorithm with the cascaded optimization strategy. The mean errors and population variances of the repeated experimental results are presented in Table 5. Ten independent calibration trials were performed on each scene, and the results are statistically summarized.

**Figure 13:**
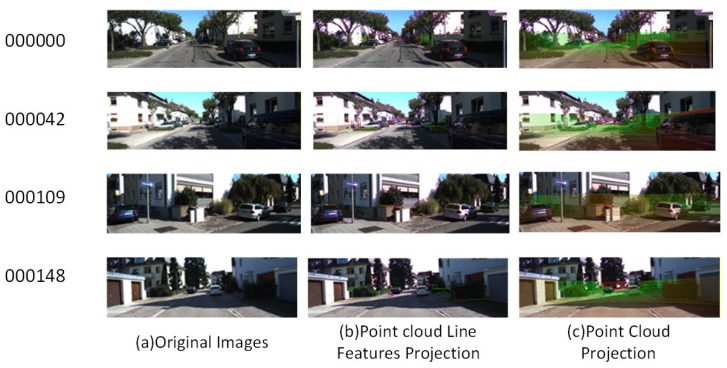
Schematic diagram of calibration results of multiple scenes in sequence 00.

Regarding the calibration results of the four sequences, they also fluctuate around the mean value, with the discrepancies remaining within the range of less than (0.03°, 0.01 m). No outlier singular values are detected. The visualization of these results is presented as follows in Figure 13.

**Figure 14:**
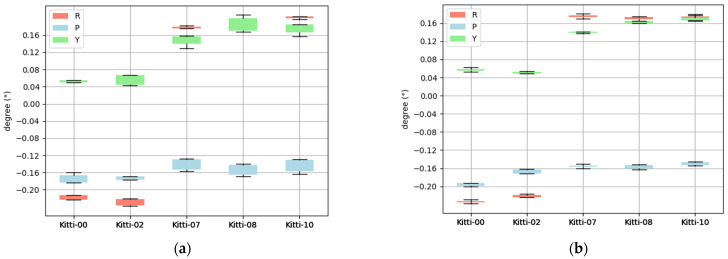
Value distribution of the convergence results of direct optimization and cascaded optimization. (**a**) Convergence of direct optimization. (**b**) Convergence of cascaded optimization.

Finally, calibrate different sequences and compare the consistency. The statistical data are as follows in Figure 14.

The nominal parts are removed from the values shown in the above figure (the 00/02 sequences are from the KITTI-2011-10-03 sequence and the 07/08/10 sequences are from the KITTI-2011-9-30 sequence, and their extrinsic parameters are slightly different). Compared to the direct optimization method, the cascaded optimization strategy achieves significantly narrowed convergence ranges for both yaw and pitch angles. This result demonstrates a pronounced enhancement in the stability of rotational parameters. In particular, the roll angle convergence for sequences 00 and 02 demonstrates moderate enhancement.

### 4.4. Convergence Efficiency

To quantitatively assess the computational efficiency and real-time capabilities of the proposed feature classification method, a comparative analysis was conducted between the cascaded optimization calibration and the direct optimization calibration. Four representative scenes from the KITTI-00 sequence were carefully selected. After the extraction of edge features, a comprehensive test of the convergence time was carried out.

The convergence condition is usually judged by two metrics: the variation in the optimized extrinsic parameters (rotation and translation) between adjacent iterations, and the decrement of the objective function (e.g., reprojection error or point cloud registration error). The iteration process stops when both the norm of the parameter update and the reduction magnitude of the error are sufficiently small.

As illustrated in Table 6, the Cascaded Optimization method incorporates an additional feature classification stage that includes quadratic fitting clustering of line-feature edges and principal direction decomposition. Despite the introduction of this extra stage, the method achieves a significant reduction in overall time consumption and a substantial acceleration in convergence speed. This is attributed to the fact that distinct features are adopted for the classified optimization of extrinsic parameters. It decomposes the original high-dimensional coupled optimization problem of rotation and translation parameters into low-dimensional subproblems. As a result, the computational complexity of core operations, such as nearest neighbor search, KD-Tree search, and SVD-based rigid transformation solving, is significantly reduced.

Meanwhile, based on the feature sensitivity selection principle, only edge features sensitive to target parameters were selected to participate in the optimization. The decoupled optimization strategy weakens the mutual interference between parameters. It renders the objective function smoother and the parameter update direction more explicit, effectively mitigating iterative oscillations.

### 4.5. Algorithm Accuracy Comparison

**Figure 15:**
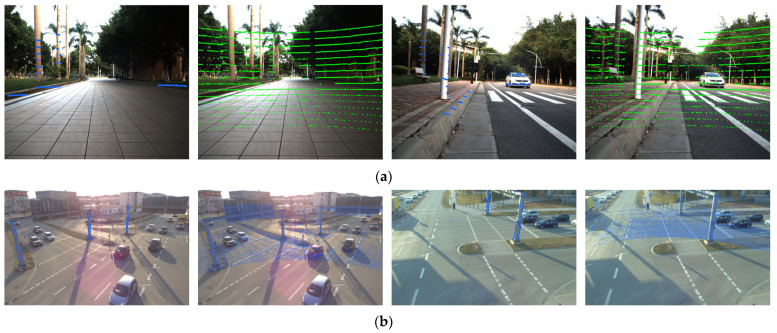
Visualization calibration results of the proposed method. (**a**) Visualization results of calibration on the S3E dataset (edge point cloud projection and all point cloud projection). (**b**) Visualization results of calibration on the TUMTraf A9 Highway Dataset (edge point cloud projection and all point cloud projection).

To evaluate the accuracy, robustness, and generalization ability of the proposed algorithm in dynamic scenarios and environments with significant illumination variations, we conducted comparative experiments against existing network-based and segmentation-based algorithms on both the S3E dataset and the TUMTraf A9 Highway Dataset. The comparative algorithms are as follows: The Calib-Anything algorithm [32] leverages the SAM network [33] and achieves training-free scene feature segmentation. Algorithm [34] matches the point cloud image semantic segmentation results through cross-domain registration and the Nelder–Mead simplex optimization algorithm. Each result shown in Table 7 is the average of ten calibration runs across multiple scenes on the dataset, and the visualization calibration results of S3E and TUMTraf A9 dataset are shown in the Figure 15.

While the network-based calibration algorithms [14,15] demonstrate superior performance on the widely adopted KITTI and S3E datasets, their heavy reliance on scenario- and viewpoint-specific pre-training severely limits their generalization ability. Due to the absence of pre-training on such viewpoints, algorithms [14,15] fail to adapt to the new observation distribution of TUMTraf A9 Highway Dataset, resulting in significant performance degradation or even calibration failure. For semantic segmentation-based extrinsic calibration algorithms, the calibration performance is closely correlated with the number of semantic features extracted from the scene. On the S3E dataset, which exhibits significant inter-scene feature heterogeneity, algorithm [32] yields lower calibration accuracy and even fails in some scenes. Meanwhile, algorithm [34] only utilizes edge information of partial semantic objects, leading to insufficient constraints and thus unstable calibration results. Despite not leveraging semantic information, the proposed algorithm extracts rich edge features from the scene and performs classification optimization. It can still stably achieve uniformly excellent calibration results on the S3E dataset with relatively sparse point clouds and the TUMTraf A9 Highway Dataset with significant dynamic interference.

Finally, the time consumption of each calibration algorithm is compared on different datasets to demonstrate the real-time performance of the algorithm, as shown in Table 8. This proves that the proposed method can achieve high-accuracy performance with less time consumption.

## 5. Discussion

This paper employs a coarse-to-fine two-stage calibration strategy to obtain the extrinsic parameters of the camera–LiDAR system. Comparative experiments with multiple mainstream algorithms and the direct optimization calibration method were conducted to verify the calibration efficiency and accuracy of the proposed cascade optimization calibration method. Meanwhile, the drawbacks of current network-based and semantic segmentation-based calibration methods in specific scenarios are briefly analyzed. In the subsequent work, we will combine theoretical tools such as the Fisher Information Matrix (FIM) and Cramér–Rao Lower Bound (CRLB) for pose estimation to quantitatively evaluate the constraint ability of edge features with different orientations on each extrinsic parameter and select the key scene features that contribute most to extrinsic calibration. On this basis, we will optimize the feature utilization mechanism, enabling efficient and accurate extrinsic calibration using only a small number of highly constraining features, thereby further breaking through the performance bottleneck of calibration in sparse-feature scenes. In addition, although Network-based calibration algorithms offer high accuracy and strong robustness, they suffer from significant limitations in cross-scene generalization and struggle to adapt to variations in sensor types (e.g., LiDARs with different channel numbers) and observation perspectives (e.g., vehicle-mounted, pedestrian, and fixed-platform setups). We plan to integrate such high-precision network-based methods with an edge-feature-based cascaded optimization approach, which exhibits superior generalization ability, to achieve stable and accurate extrinsic estimation under diverse scenes and working conditions.

## 6. Conclusions

Traditional automatic online targetless calibration methods often suffer from poor scene adaptability and the unstable convergence of extrinsic parameters. To tackle these challenges, this paper presents an innovative online calibration approach for camera–LiDAR extrinsic parameters. The proposed method comprehensively analyzes the motion and geometric observation characteristics of both the camera and LiDAR. The method leverages cascade optimization of motion trajectories and edge features, enabling the real-time calculation of extrinsic parameters in complex scenes without relying on artificial markers. Experimental verification using typical urban datasets demonstrate that the proposed method achieves high accuracy and real-time performance, validating its effectiveness in practical applications.

## Figures and Tables

**Table 1:** Initial calibration error.

Dataset	Initial Calibration Error (deg)		
	Roll	Pitch	Yaw
Kitti-00	1.00032	1.613806	1.273831
Kitti-02	1.48803	1.247043	1.71953
Kitti-07	0.83487	1.21678	1.782552
Kitti-08	0.85356	1.56082	1.419829
Kitti-10	0.995012	1.228043	1.321027
Average	1.034358	1.373298	1.503354

**Table 2:** Calibration errors of different optimization methods.

Seq.	Calibration Error of Direct Optimization (deg)			Calibration Error of Cascaded Optimization (deg)		
	Roll	Pitch	Yaw	Roll	Pitch	Yaw
Kitti-00	0.1707667	0.389077	0.3632944	0.1233405	0.2179646	0.1628424
Kitti-02	0.1676692	0.391413	0.3609313	0.1359476	0.2460872	0.15339422
Kitti-07	0.122317	0.271207	0.283961	0.1430422	0.2305978	0.14178701
Kitti-08	0.196411	0.390769	0.3972172	0.1368827	0.2286838	0.1600889
Kitti-10	0.209961	0.401655	0.3523188	0.1379462	0.2358834	0.1693664
Average	0.1734250	0.368824	0.3515445	0.1354318	0.2318434	0.1574958

**Table 3:** Calibration error in the same scene.

Kitti-01-000102	Roll (deg)	Pitch (deg)	Yaw (deg)	T (m)
1	0.1255785	0.2116630	0.1986781	0.034
2	0.1004556	0.1972776	0.2113779	0.042
3	0.1156924	0.2152960	0.1890919	0.036
4	0.1214647	0.2081278	0.1882282	0.038
5	0.1073971	0.2047583	0.1851614	0.044
Average	0.11411766	0.20742454	0.1945075	0.0388

**Table 4:** Calibration result errors of multiple scenes in sequence 00.

Kitti-00 Sequence	Roll (deg)	Pitch (deg)	Yaw (deg)	X (m)	Y (m)	Z (m)
000000	0.1309638	0.2257980	0.1504200	0.017	0.024	0.029
000042	0.12597018	0.22621772	0.15258788	0.016	0.025	0.030
000109	0.1048144	0.2208563	0.1699648	0.018	0.026	0.032
000148	0.11580874	0.22639874	0.16405685	0.019	0.026	0.032

**Table 5:** Repeated calibration experiments.

Kitti-00 Sequence	Mean Error		Population Variance	
	Rotation (deg)	Translation (m)	Rotation (deg^2^)	Translation (m^2^)
000000	0.213	0.0421	8.97 × 10^−4^	3.78 × 10^−4^
000042	0.206	0.0407	7.09 × 10^−4^	4.33 × 10^−4^
000109	0.231	0.0479	6.28 × 10^−4^	2.41 × 10^−4^
000148	0.244	0.0431	3.44 × 10^−4^	2.52 × 10^−4^

**Table 6:** Time consumption of direct optimization and cascaded optimization.

Kitti-00 Sequence	Direct Optimization Calibration (s)	Cascaded Optimization Calibration (s)		
		Feature Classification Time	Optimization Time	Total Calibration Time
00000	29.63	0.94	17.11	18.05
00042	29.17	0.91	17.82	18.73
00109	27.33	0.88	16.69	17.57
00148	28.16	0.89	17.52	18.41
Average	28.572	0.905	17.285	18.19

**Table 7:** Accuracy comparison of various methods.

L2 Loss		Networks-Based		Semantic Segmentation-Based		Ours
		[14]	[15]	[32]	[34]	
KITTI	Rotation (deg)	0.223	0.244	**0.212**	0.340	0.210
	Translation (m)	0.0687	0.0699	0.1070	0.2020	**0.0411**
S3E	Rotation (deg)	0.307	**0.221**	0.445	-	0.231
	Translation (m)	0.0811	0.0864	0.2010	-	**0.0571**
TUMTraf A9	Rotation (deg)	1.070	-	0.297	0.314	**0.181**
	Translation (m)	0.675	-	0.210	0.10	**0.0723**

Note: 1. Bold data is the optimal. 2. “-” represents calibration failure.

**Table 8:** Time consumption of different methods.

Time Consumption (s)	[14]	[15]	[32]	[34]	Ours
KITTI	101.55	-	44.11	60.22	26.0
S3E	94.77	-	30.56	-	22.22
TUMTraf A9	100.21	-	31.93	44.50	18.94

Note: 1. Average time consumption over ten calibration runs across multiple scenes. 2. Reference [15] requires processing the entire dataset sequentially, and thus does not support real-time operation.

## Data Availability

The original contributions presented in the study are included in the article, further inquiries can be directed to the corresponding author.

## Appendix A

**Algorithm A1.** Processing Point Clouds via Euclidean Clustering and PCA Decomposition.
**Input**: Point cloud data matrix P, clustering tolerance CT, minimum cluster size MCS, maximum cluster size XCS **Output:** Point cloud line features LineFeature_z, LineFeature_xy, LineFeature_other, classification results 1: Initialize point cloud P 2: Initialize KD—tree KDTree 3: Set input point cloud KDTree as P 4: Initialize Euclidean clustering object EC 5: Set clustering tolerance EC.ct→CT 6: Set minimum cluster size EC.mcs→MCS 7: Set maximum cluster size EC.xcs→XCS 8: Set search method EC.method→KDTree 9: Set input point cloud EC.input→P 10: Execute clustering EC, get clustering results clusters 11: Initialize dictionary P 12: For each cluster cluster∈clusters do: 13: Initialize the point-set points of the current cluster 14: For index∈cluster do: 15: points.push_back(P[index]) 16: End loop 17: Initialize PCA object 18: Perform PCA on points, get the principal axis direction direction 19: Calculate the angle θ between the principal axis direction direction and the X−Y plane 20: IF θ≥UpperThreshold THEN 21: do LineFeature_z.push_back(points); 22: ELSE θ≤LowerThreshold THEN 23: do LineFeature_xy.push_back(points); 24: ELSE 25: do LineFeature_other.push_back(points) 26: END IF 27: END LOOP 28: Return LineFeature_z,LineFeature_xy,LineFeature_other
